# Draft Genome Sequence of Multidrug-Resistant *Acinetobacter baumannii* Strain MMC4, Isolated from a Patient in Tennessee

**DOI:** 10.1128/genomeA.00051-14

**Published:** 2014-02-20

**Authors:** Sammed N. Mandape, Dana R. Marshall, L. Leon Dent, Siddharth Pratap

**Affiliations:** a Department of Microbiology and Immunology, Meharry Medical College, Nashville, Tennessee, USA; b Department of Pathology, Anatomy and Cell Biology, Meharry Medical College, Nashville, Tennessee, USA; c Department of Surgery, Meharry Medical College, Nashville, Tennessee, USA; d School of Graduate Studies and Research, Meharry Medical College, Nashville, Tennessee, USA

## Abstract

*Acinetobacter baumannii* multidrug-resistant strain MMC4 was isolated from a bronchoalveolar lavage fluid sample from a patient in Nashville, TN, USA. Here, we report a draft genome sequence with a size of 3,985,367 bp, an average G+C content of 39.8%, and 3,863 predicted protein-coding sequences.

## GENOME ANNOUNCEMENT

*Acinetobacter baumannii*, a nonfermenter of glucose, is an aerobic Gram-negative rod. It causes severe nosocomial infections and has been shown to increase mortality and length of hospital stay (1). Outbreaks of infection caused by *A. baumannii* have been increasingly reported since 2000 (2–5). Additionally, this bacterium can acquire foreign DNA through lateral gene transfer, thus facilitating multidrug resistance and pathogenicity (6). Resistance in this organism has rapidly increased in a very short time frame. *A. baumannii* has also been identified as a member of the ESKAPE pathogens (*Enterococcus faecium, Staphylococcus aureus, Klebsiella pneumoniae, Acinetobacter baumannii, Pseudomonas aeruginosa*, and *Enterobacter* species), a group of pathogens with a high rate of antibiotic resistance that are responsible for a majority of nosocomial infections (7).

In this study, we report a draft genome sequence of the *A. baumannii* multidrug-resistant strain MMC4, isolated from a bronchoalveolar lavage fluid specimen from a patient at Nashville General Hospital (NGH), Nashville, TN (8). Whole-genome shotgun sequencing was carried out with 43-bp single-end reads on an Illumina Genome Analyzer IIx and with 250-bp paired-end reads (with 2k spacers) on a Roche 454 GS FLX. *De novo* assembly was done using SOAP*denovo* for the Illumina reads and the Newbler assembler for the 454 reads. A hybrid assembling strategy yielded a draft genome of *A. baumannii* MMC4 consisting of 148 contigs, with a total length of 3,985,367 bp, an N_50_ of 66,205 bp, and a mean contig length of 26,928 bp. The overall G+C content was determined to be 39.8%. The final draft sequence consists of 146 contigs.

Open reading frame (ORF) prediction was conducted with Glimmer version 3.02 (9), resulting in 4,039 predicted ORFs in the draft genome. The RAST server (10) was used to annotate the contigs, resulting in 3,863 predicted protein-coding genes, with an average length of 901 bp. The total length of the coding sequences (CDS) is 3,481,467 bp, covering 87.35% of the draft genome. We also found 63 tRNA genes, with a total length of 4,721 bp, covering 0.1185% of the draft genome. In addition, 2 rRNAs and 1 small RNA (sRNA) were detected.

### Nucleotide sequence accession numbers.

This whole-genome shotgun project has been deposited at DDBJ/EMBL/GenBank under the accession no. AZNQ00000000. The version described in this paper is the first version, AZNQ01000000.
